# Supplementary figures and images for: Gle1 is required for tRNA to stimulate Dbp5 ATPase activity in vitro and promote Dbp5-mediated tRNA export in vivo in Saccharomyces cerevisiae
Source: eLife. 2024 Jan 8;12:RP89835. doi: 10.7554/eLife.89835 (PMC10945473; doi:10.7554/eLife.89835)

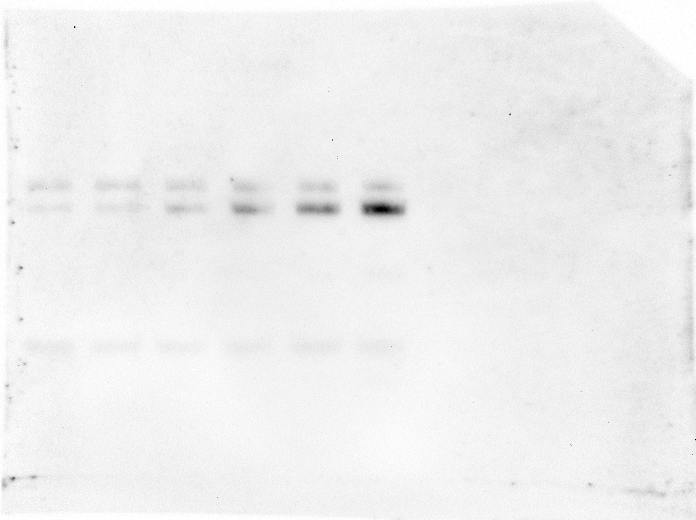

Supplement: Figure 1—source data 1. [file elife-89835-fig1-data1.zip › Figure 1-source data 1_raw data files for Northern Blots/Figure 1F Source data_Ile UAU.tif]

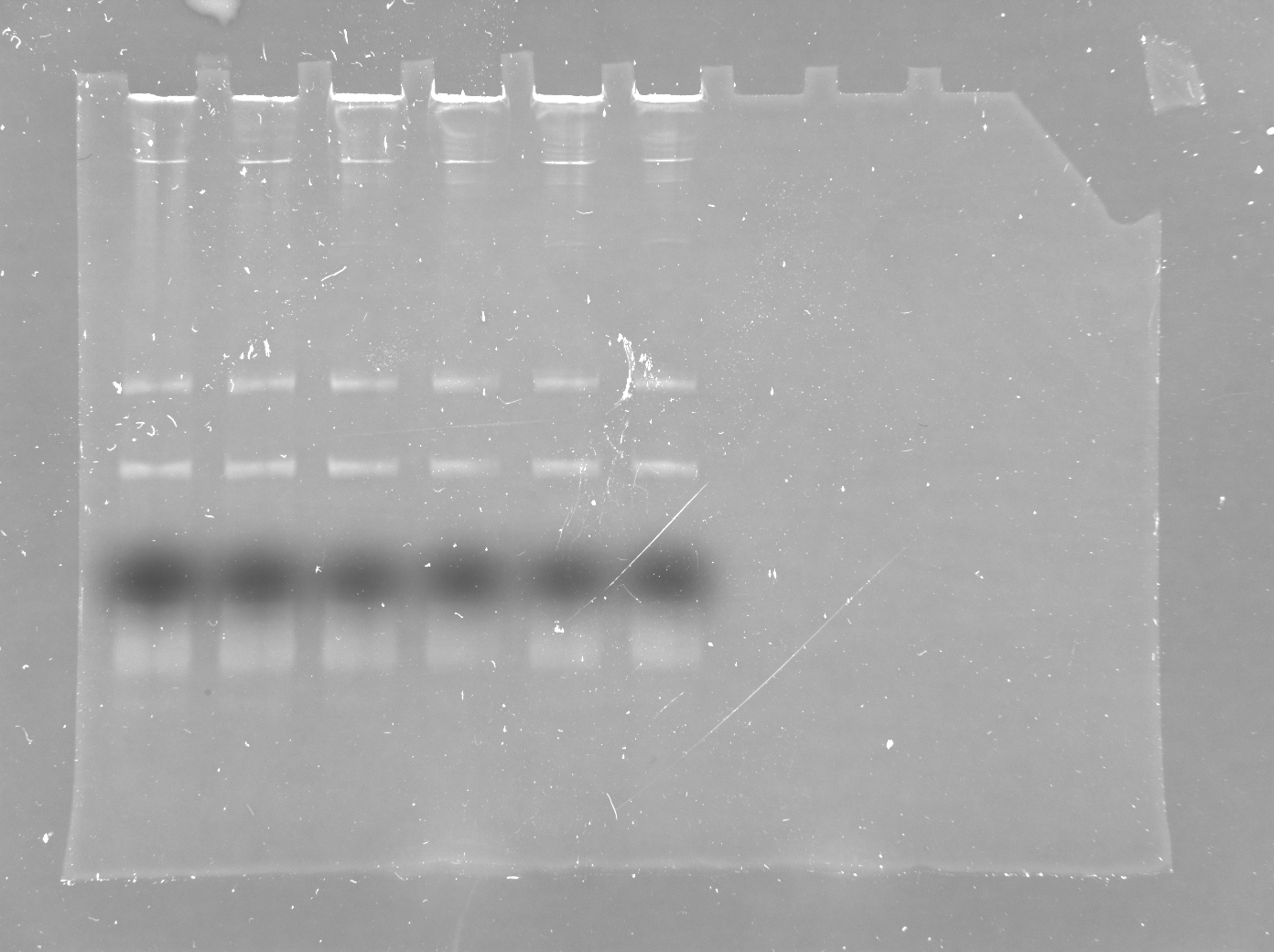

Supplement: Figure 1—source data 1. [file elife-89835-fig1-data1.zip › Figure 1-source data 1_raw data files for Northern Blots/Figure 1F Source data_ApexStain.tif]

Figure 1F

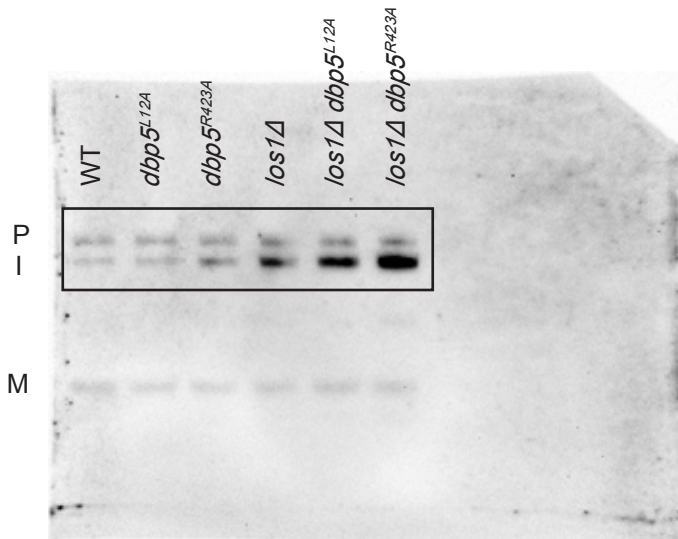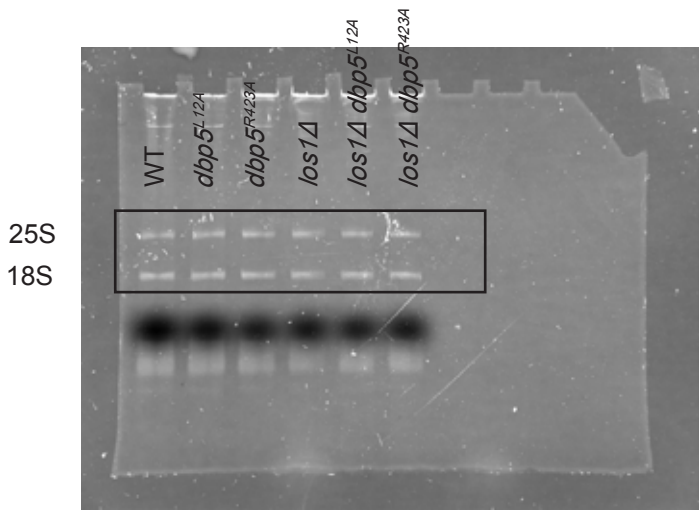

Supplement: Figure 1—source data 2. [file elife-89835-fig1-data2.zip › Figure 1-source data 2_annotated Northern Blots/Figure1-source data 2.pdf]

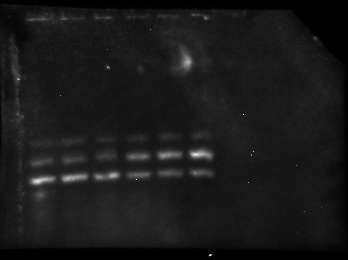

Supplement: Figure 1—figure supplement 1—source data 1. [file elife-89835-fig1-figsupp1-data1.zip › Figure 1-figure supplement 1 source data 1_raw data files for Northern Blots/Figure 1-figure supplement 1E source data 1_Tyr GUA.tif]

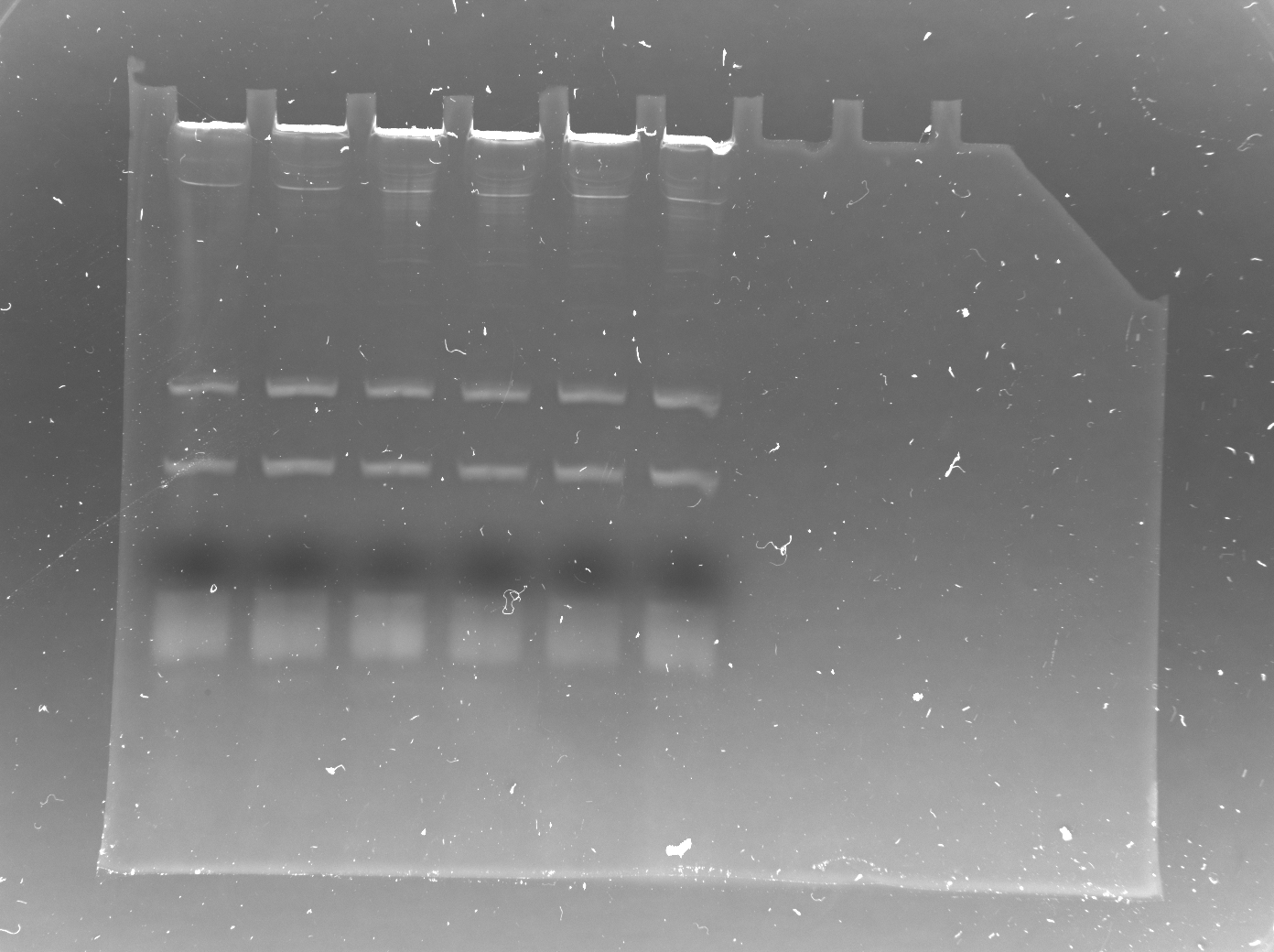

Supplement: Figure 1—figure supplement 1—source data 1. [file elife-89835-fig1-figsupp1-data1.zip › Figure 1-figure supplement 1 source data 1_raw data files for Northern Blots/Figure 1-figure supplement 1E source data 1_ApexStain.tif]

Figure 1-figure supplement 1E

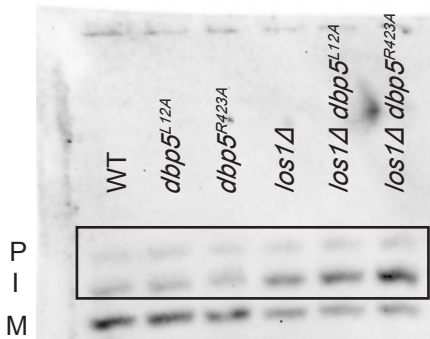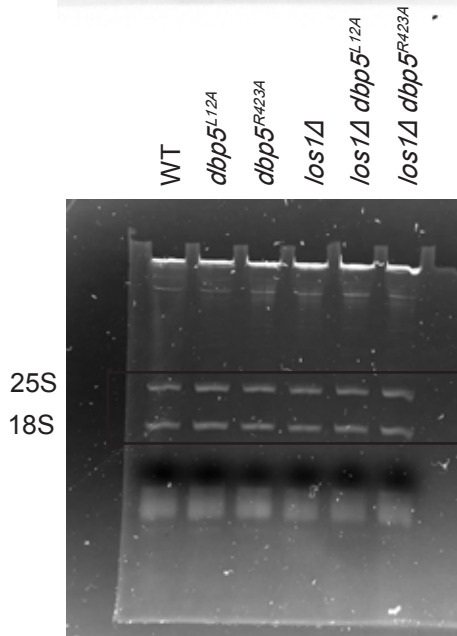

Supplement: Figure 1—figure supplement 1—source data 2. [file elife-89835-fig1-figsupp1-data2.zip › Figure 1-figure supplement 1 source data 2_annotated Northern Blots/Figure1-figure supplement 1-source data 2.pdf]

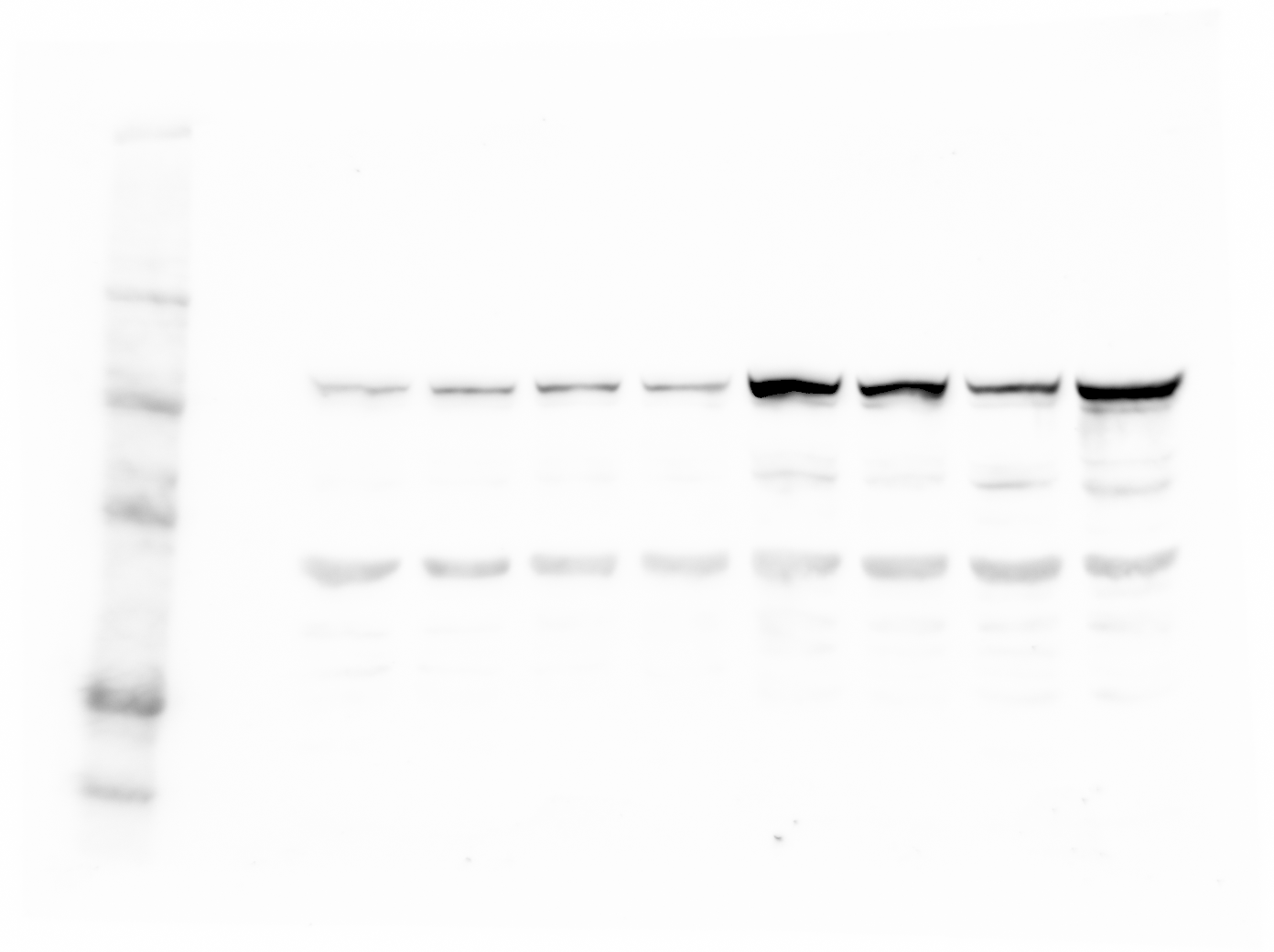

Supplement: Figure 3—source data 1. [file elife-89835-fig3-data1.zip › Figure 3-source data 1_raw data files for Western and Northern Blots/Figure 3A-source data 1_DBP5 and GAPDH Western.tif]

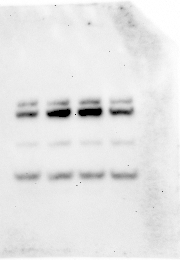

Supplement: Figure 3—source data 1. [file elife-89835-fig3-data1.zip › Figure 3-source data 1_raw data files for Western and Northern Blots/Figure 3C-source data 1_Ile UAU Northern.tif]

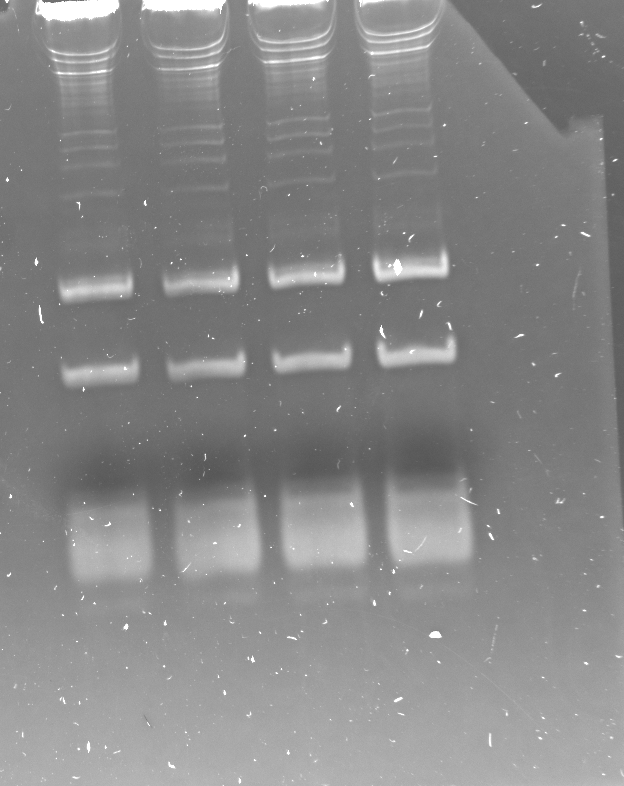

Supplement: Figure 3—source data 1. [file elife-89835-fig3-data1.zip › Figure 3-source data 1_raw data files for Western and Northern Blots/Figure 3C-source data 1_Ile UAU Northern_ApexStain.tif]

Figure 3A

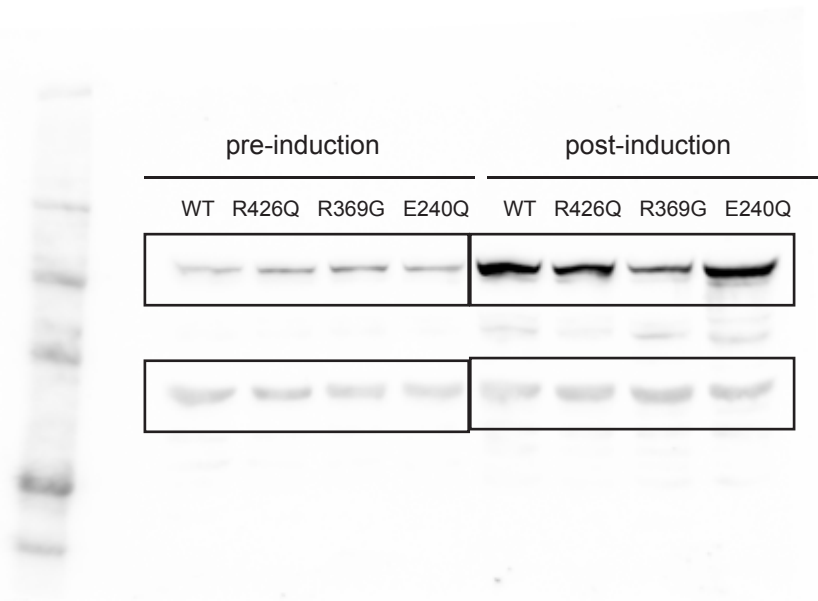

Figure 3C

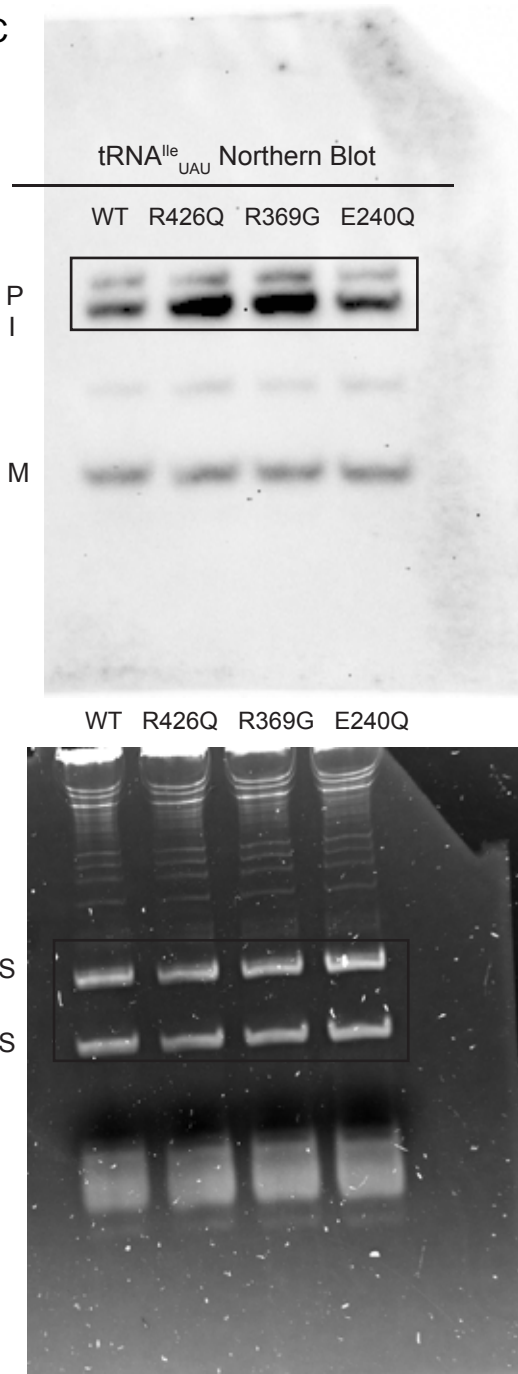

Supplement: Figure 3—source data 2. [file elife-89835-fig3-data2.zip › Figure 3-source data 2_annotated Western and Northern Blots/Figure3-source data 2.pdf]

Figure 4A

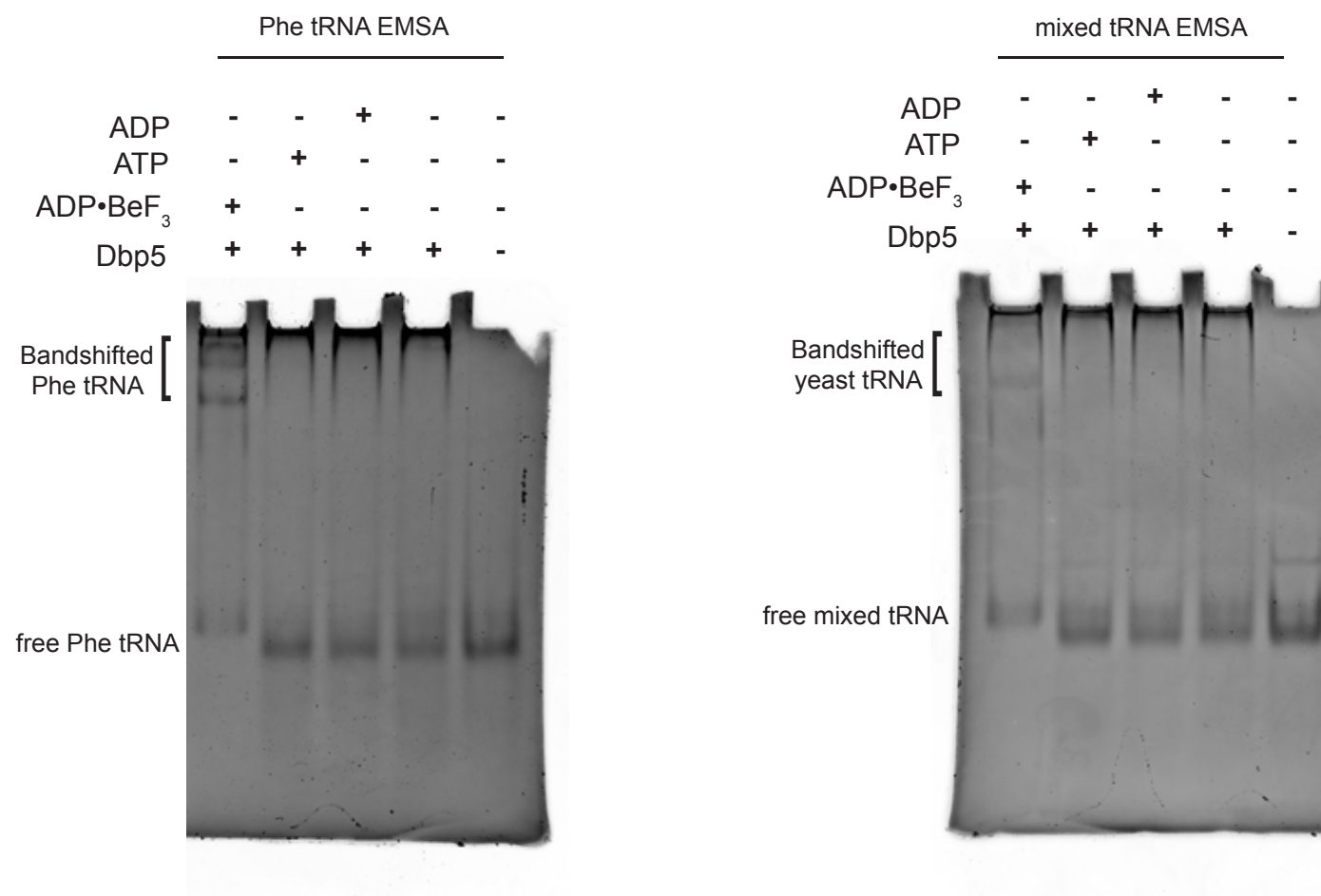

Figure 4B

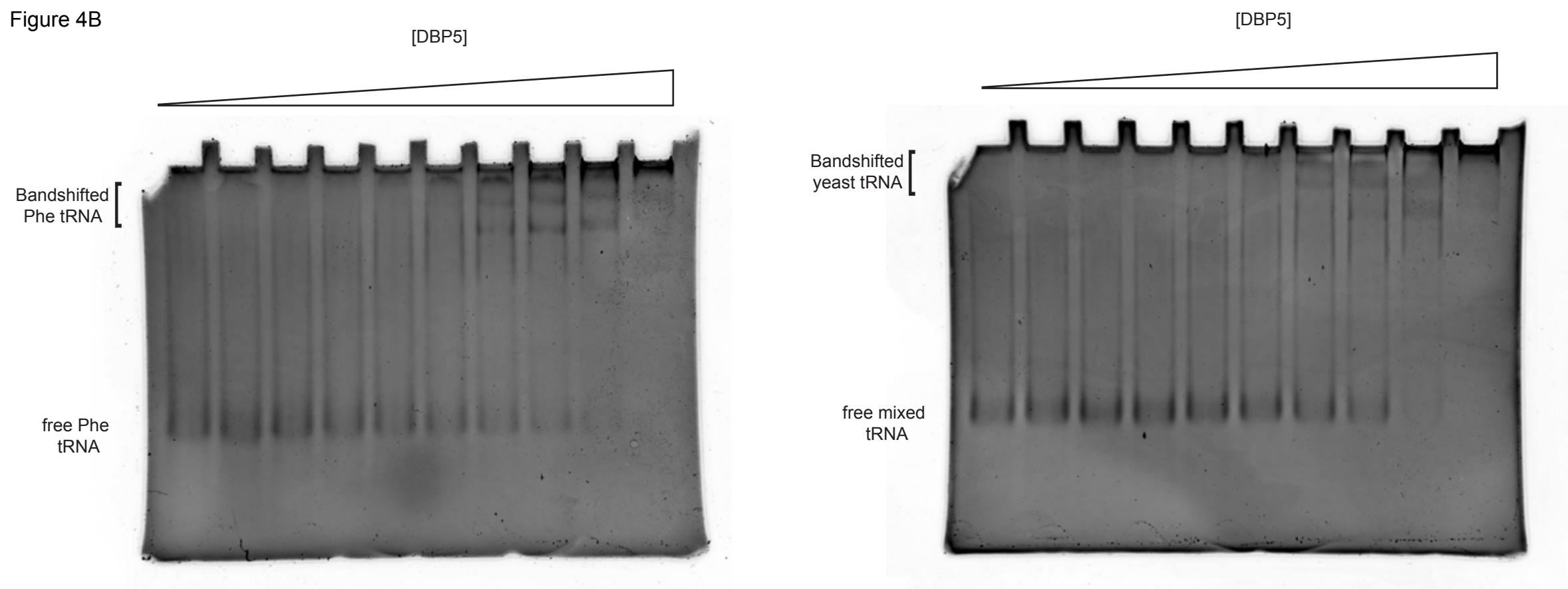

Supplement: Figure 4—source data 1. [file elife-89835-fig4-data1.zip › Figure 4-source data 2_annotated EMSAs/Figure 4-source data 2.pdf]

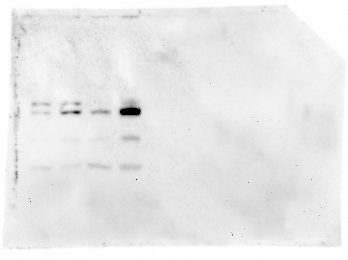

Supplement: Figure 5—source data 1. [file elife-89835-fig5-data1.zip › Figure 5-source data 1_raw data files for Northern Blots/Figure 5D-source data 1_Ile UAU.tif]

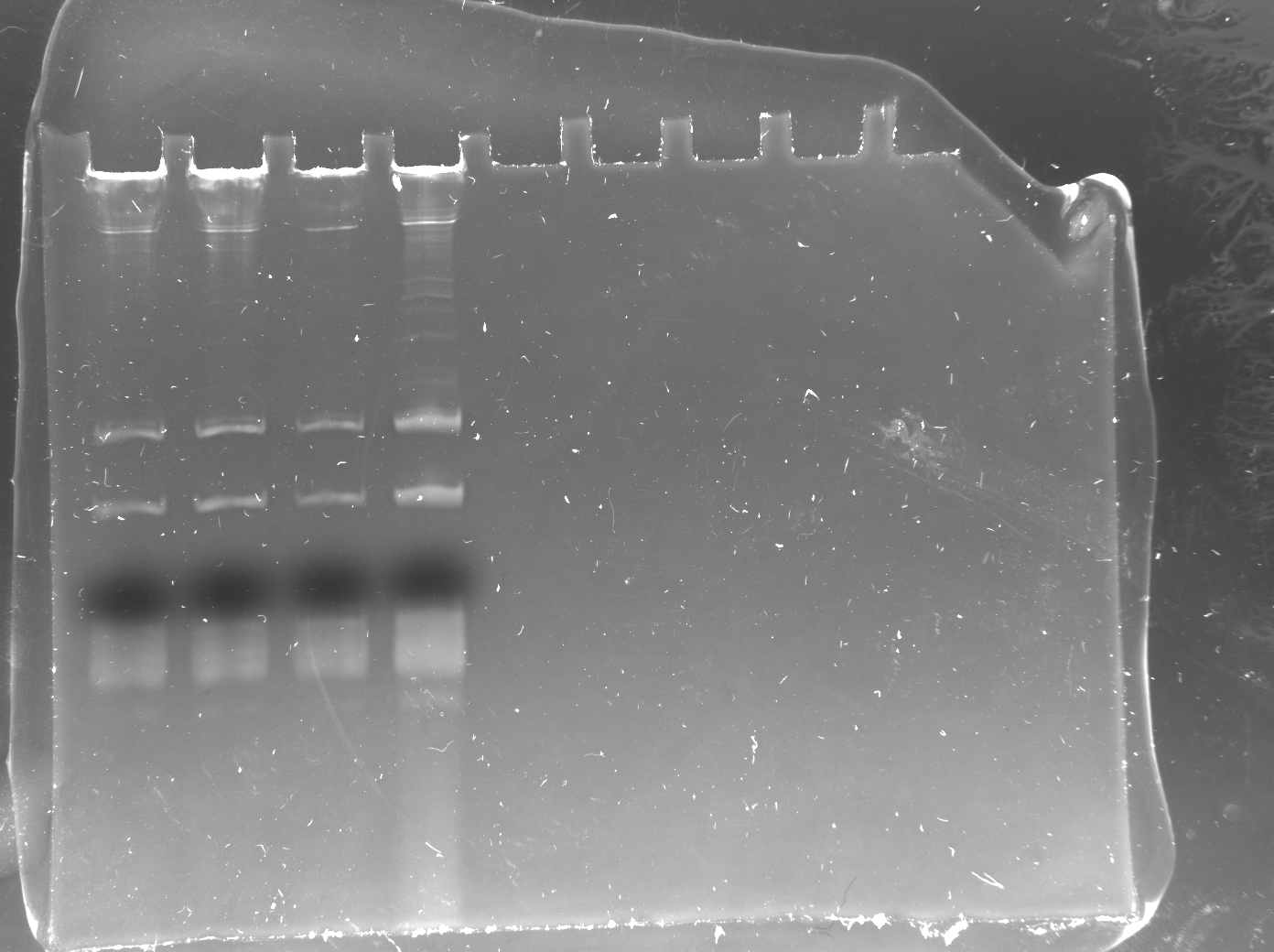

Supplement: Figure 5—source data 1. [file elife-89835-fig5-data1.zip › Figure 5-source data 1_raw data files for Northern Blots/Figure 5D-source data 1_ApexStain.tif]

Figure 5D

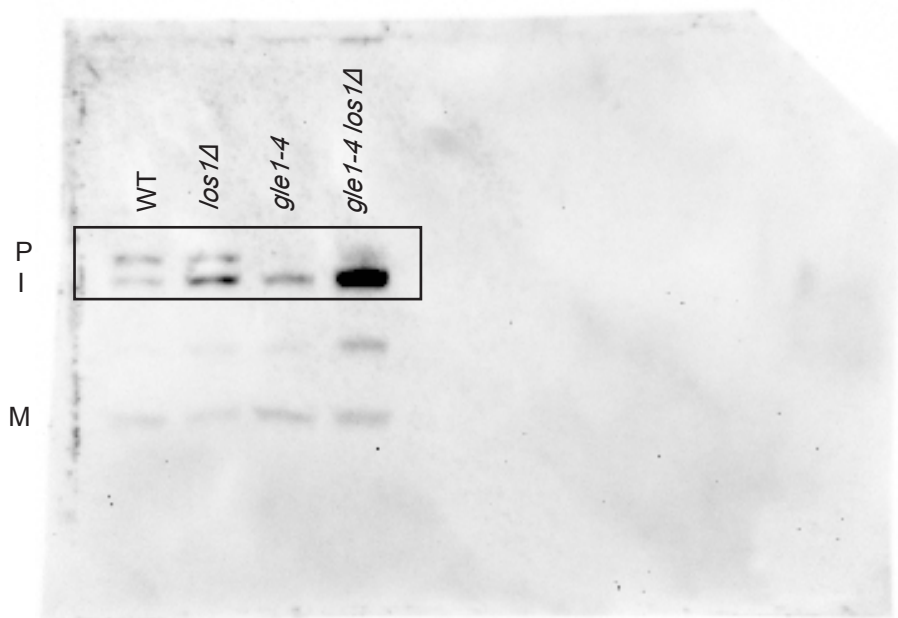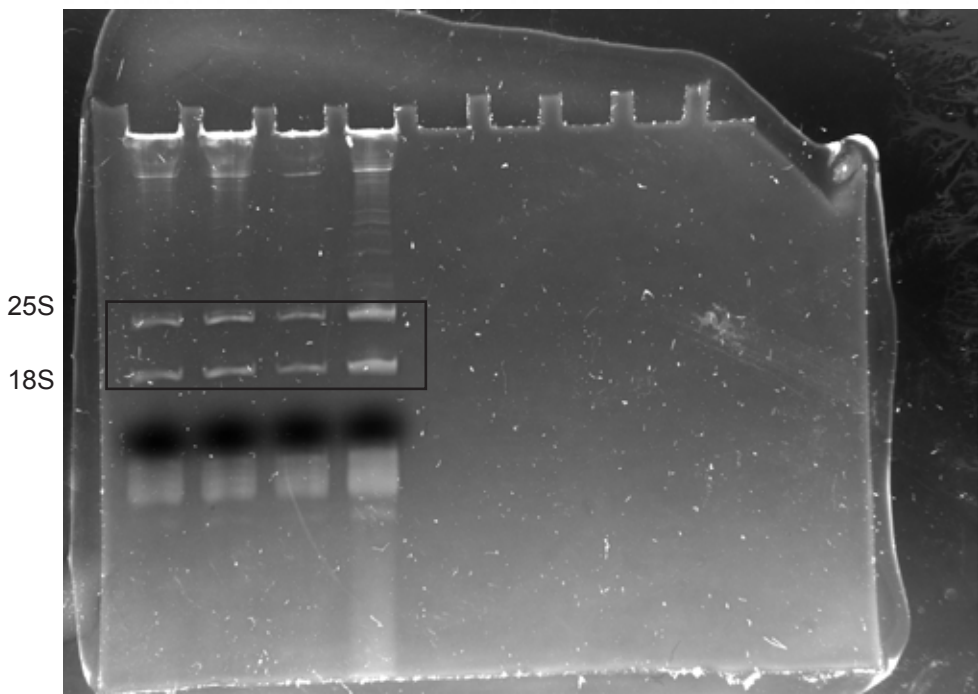

Supplement: Figure 5—source data 2. [file elife-89835-fig5-data2.zip › Figure 5-source data 2_annotated Northern Blots/Figure5-source data 2.pdf]
